# ﻿Characterization of the complete mitochondrial genome of the longhorn beetle, *Batocerahorsfieldi* (Coleoptera, Cerambycidae) and its phylogenetic analysis with suitable longhorn beetles

**DOI:** 10.3897/zookeys.1168.105328

**Published:** 2023-07-04

**Authors:** Junhao Wu, Danping Xu, Xinju Wei, Wenkai Liao, Xiushan Li, Zhihang Zhuo

**Affiliations:** 1 College of Life Science, China West Normal University, Nanchong, 637002, China China West Normal University Nanchong China

**Keywords:** Evolutionary relationships, mitogenome, pest species, protein-coding genes, ribosomal RNA genes, secondary structure, tRNA

## Abstract

Mitochondrial genome analysis is an important tool for studying insect phylogenetics. The longhorn beetle, *Batocerahorsfieldi*, is a significant pest in timber, economic and protection forests. This study determined the mitochondrial genome of *B.horsfieldi* and compared it with the mitochondrial genomes of other Cerambycidae with the aim of exploring the phylogenetic status of the pest and the evolutionary relationships among some Cerambycidae subgroups. The complete mitochondrial genome of *B.horsfieldi* was sequenced by the Illumina HiSeq platform. The mitochondrial genome was aligned and compared with the existing mitochondrial genomes of *Batoceralineolata* and *B.rubus* in GenBank (MF521888, MW629558, OM161963, respectively). The secondary structure of transfer RNA (tRNA) was predicted using tRNAScan-SE server v.1.21 and MITOS WebSever. Thirteen protein-coding genes (PCGs) and two ribosomal RNA gene sequences of 21 longhorn beetles, including *B.horsfieldi*, plus two outgroups, *Dryopsernesti* (Dryopidae) and *Heterocerusparallelus* (Heteroceridae), were analyzed. The phylogenetic tree was constructed using maximum likelihood and Bayesian inference methods. In this study, we successfully obtained the complete mitochondrial genome of *B.horsfieldi* for the first time, which is 15 425 bp in length. It contains 37 genes and an A + T-rich region, arranged in the same order as the recognized ancestor of longhorn beetles. The genome of *B.horsfieldi* is composed of 33.12% A bases, 41.64% T bases, 12.08% C bases, and 13.16% G bases. The structure, nucleotide composition, and codon usage of the new mitochondrial genome are not significantly different from other longhorn mitochondrial genomes. Phylogenetic analyses revealed that Cerambycidae formed a highly supported single clade, and Vesperidae was either clustered with Cerambycidae or formed a separate clade. Interestingly, *B.horsfieldi*, *B.rubus* and *B.lineolata* were clustered with *Monochamus* and *Anoplophora* species in both analyses, with high node support. Additionally, the Vesperidae*Spiniphilusspinicornis* and *Vesperussanzi* and the 19 Cerambycidae species formed a sister clade in the Bayesian analysis. Our results have produced new complete mitogenomic data, which will provide information for future phylogenetic and taxonomic research, and provide a foundation for future relevant research.

## ﻿Introduction

The longhorn beetle family Cerambycidae is one of the larger families in Coleoptera, with about 40 000 recorded species worldwide. As of 2005, the recorded number of longhorn beetle species in China had surpassed 3100 (Zhang 2011). Many of the Cerambycidae are notorious pests, as they damage crops, forests, orchards and garden plants. *Batocerahorsfieldi* (Hope, 1839) is a significant pest in timber, economic and protection forests. This species is mainly distributed in Asian countries such as China, Vietnam, India, Myanmar, etc. (Xiao 1992). It has a wide range of host plants, including *Populustomentosa* Carr., *Juglansregia* L., *Eucalyptusrobusta* Sm. (Zhuo et al. 2022). Adults of *B.horsfieldi* fed on the tender bark and leaves of supplemental host plants, such as *Rosacymosa* Tratt., *Rosamultiflora* Thunb., *Fraxinuschinensis* Roxb. (Xiao 1992), while larvae bore into the trunk and feed on the woody tissue of the host plants. Larvae of *B.horsfieldi* burrow inside tree trunks, causing the host’s xylem to decay and become hollow. The entry holes are blocked by sawdust and excrements, making it difficult to control with pesticide. Due to their biological habits, larvae are prone to causing host tree weakness, fungal infections, wind breaks and even the death of the entire host tree (Zhuo et al. 2022).

The mitochondrial genome is widely used in population genetics, phylogenetics and molecular evolution studies due to its maternal inheritance, stable genome composition, relatively conservative gene arrangement, and rare occurrence of recombination (Wolstenholme 1992; Nelson et al. 2012; Driscoll et al. 2015; Feng et al. 2015). With the development of next-generation sequencing technology in the mitochondrial genome, it is possible to obtain more molecular markers for phylogenetic analysis, resulting in more robust relationships (Li et al. 2015; Fagua et al. 2018). Tracking the expansion pathways and dynamic hybridization of arthropods is often accomplished through mitochondrial sequence analysis (Rubinoff et al. 2010; Moore et al. 2013; Wang et al. 2017). The mitochondrial genome is widely used for phylogenetic inference in animals from high to low taxonomic levels (Li et al. 2016; Zheng et al. 2018), including tribe-level (Nie et al. 2018), subfamily-level (Nie et al. 2020) and family-level (Nie et al. 2021) relationships of beetles. To date, only seven species of *Batocera* have had their mitochondrial genomes sequenced. Therefore, this study reports the first complete mitochondrial genome sequence of *B.horsfieldi* using second-generation sequencing technology and compares it with the 20 existing mitochondrial genome sequences of other longhorn beetles in GenBank. In addition, we inferred the phylogenetic relationships of 21 longhorn beetle species to better study the phylogeny of Cerambycidae. The establishment of this genome resource will provide information for future phylogenetic and taxonomic research, providing a foundation for future relevant research.

## ﻿Material and methods

### ﻿Sample collection and DNA extraction

The specimens of *B.horsfieldi*, three male adults and one female adult, were collected from the host plant *Populustomentosa* in Xishan Forest Park, Nanchong City, Sichuan Province, China, on June 17, 2022 (30.803038°N, 106.063072°E, alt. 480 m), and were deposited in the Laboratory of Forest Conservation, College of Life Science, China West Normal University (Voucher No. SCNC-BH-20220617.1-3 for male adult, SCNC-BH-20220617.4 for female adult). These specimens were preserved in 95% ethanol at -24 °C for long-term storage in the specimen collection room at China West Normal University. The total genomic DNA was extracted from muscle tissue of individual specimens using the Ezup Column Animal Genomic DNA Purification Kit (Shanghai, China), following the manufacturer’s instructions. For sequencing, the extracted DNA was stored at -24 °C.

### ﻿DNA sequencing, mitogenome assembly and annotation

Next-generation sequencing and assembly were performed by Beijing Aoweisen Gene Technology Co. Ltd (Beijing, China) to obtain the complete mitogenome of *B.horsfieldi* for the first time. After quantification of the total genome DNA, a whole-genome shotgun strategy was employed for sequencing on the Illumina HiSeq platform. A total of 14 348 102 paired-end reads with a read length of 150 bp were obtained from the sequencing process. Among these, 22 966 sequences were used for mitochondrial genome assembly. Based on the invertebrate genetic code, such as *Batoceralineolata* Chevrolat, 1852 (MF521888), *B.lineolata* (MW629558) and *B.rubus* Linnaeus, 1758 (OM161963), the assembly of *B.horsfieldi* was carried out by referring to Hahn et al. (2013), and the gene annotation was performed using Geneious v.11.0.2 by referring to Greiner et al. (2019). The tRNA genes were re-verified by tRNAScan-SE (Lowe and Eddy 1997) and MITOS WebSever (Bernt et al. 2013). The base composition and relative synonymous codon usage (RSCU) values were calculated using Shengxin Cloud Online Server. Strand asymmetry was calculated in terms of formulae: AT-skew = (A - T)/(A + T) and GC-skew = (G - C)/(G + C) (Perna and Kocher 1995).

### ﻿Phylogenetic analyses

A total of 21 complete mitochondrial genomes of longhorn beetles, including one newly sequenced species (*B.horsfieldi*), as well as the complete mitochondrial genomes of *Dryopsernesti* DesGozis, 1886 and *Heterocerusparallelus* Gebler, 1830, were used in this study (Table 1). These species belong to 13 longhorn genera, with *Heterocerusparallelus* (Heteroceridae) and *Dryopsernesti* (Dryopidae) as outgroups. The utilization of tRNA genes in phylogenetic analysis is relatively infrequent due to their short length and highly conserved characteristics (Chen 2005), thus we constructed the phylogenetic tree based on the 13 PCGs and rRNAs by using PhyloSuite v.1.4.4, and analyzed the tree by maximum likelihood (ML) and Bayesian inference (BI) methods with different best-fit substitution models. The sequences were aligned using MAFFT(Multiple Alignment using Fast Fourier Transform)and trimmed by trimAl. The best-fit substitution models used in the ML and BI analyses were calculated using ModelFinder v.2.3. Maximum likelihood analyses were run with 1000 ultrafast bootstrap and 1000 SH-aLRT replicates to estimate node reliability. Bayesian analyses were run with two independent chains spanning 1 million generations, four Markov chains, sampling at every 100 generations, and with a burn-in of 25%. The phylogenetic tree was visualized and edited using the iTOL online server (https://itol.embl.de/).

**Table 1.** GenBank accession numbers of species used in this study (accession number of the newly sequenced species in bold).

Family	Genus	Species	GenBank Accession number
Cerambycidae	*Monochamus*	*Monochamussparsutus*	NC053906.1
		*Monochamusalternatus*	NC050066.1
		*Monochamussartorurussovii*	OP856519.1
		*Monochamussaltuarius*	OP169419.1
	*Agapanthia*	*Agapanthiaamurensis*	MW617354.1
		*Agapanthiadaurica*	MN473114.1
	*Anoplophora*	*Anoplophoraglabripennis*	NC008221.1
		*Anoplophorahorsfieldi*	MW364565.1
		*Anoplophorachinensis*	NC029230.1
	*Batocera*	*Batoceralineolata*	MW629558.1
		*Batocerarubus*	OM161963.1
		*Batocerahorsfieldi*	**OQ785650**
	*Xylotrechus*	*Xylotrechusgrayii*	NC030782.1
	*Turanoclytus*	*Turanoclytusnamaganensis*	NC060874.1
	*Demonax*	*Demonaxpseudonotabilis*	OP096419.1
	*Allotraeus*	*Allotraeusorientalis*	NC061181.1
	*Arhopalus*	*Arhopalusunicolor*	NC053904.1
	*Aromia*	*Aromiabungii*	NC053714.1
	*Cephalallus*	*Cephalallusoberthueri*	NC062854.1
Vesperidae	*Vesperus*	*Vesperussanzi*	MN473093.1
	*Spiniphilus*	*Spiniphilusspinicornis*	NC029515.1
Out groups	*Dryops*	*Dryopsernesti*	KX035147.1
	*Heterocerus*	*Heterocerusparallelus*	KX087297.1

## ﻿Results

### ﻿Mitogenome organization and composition

**Figure 1.**
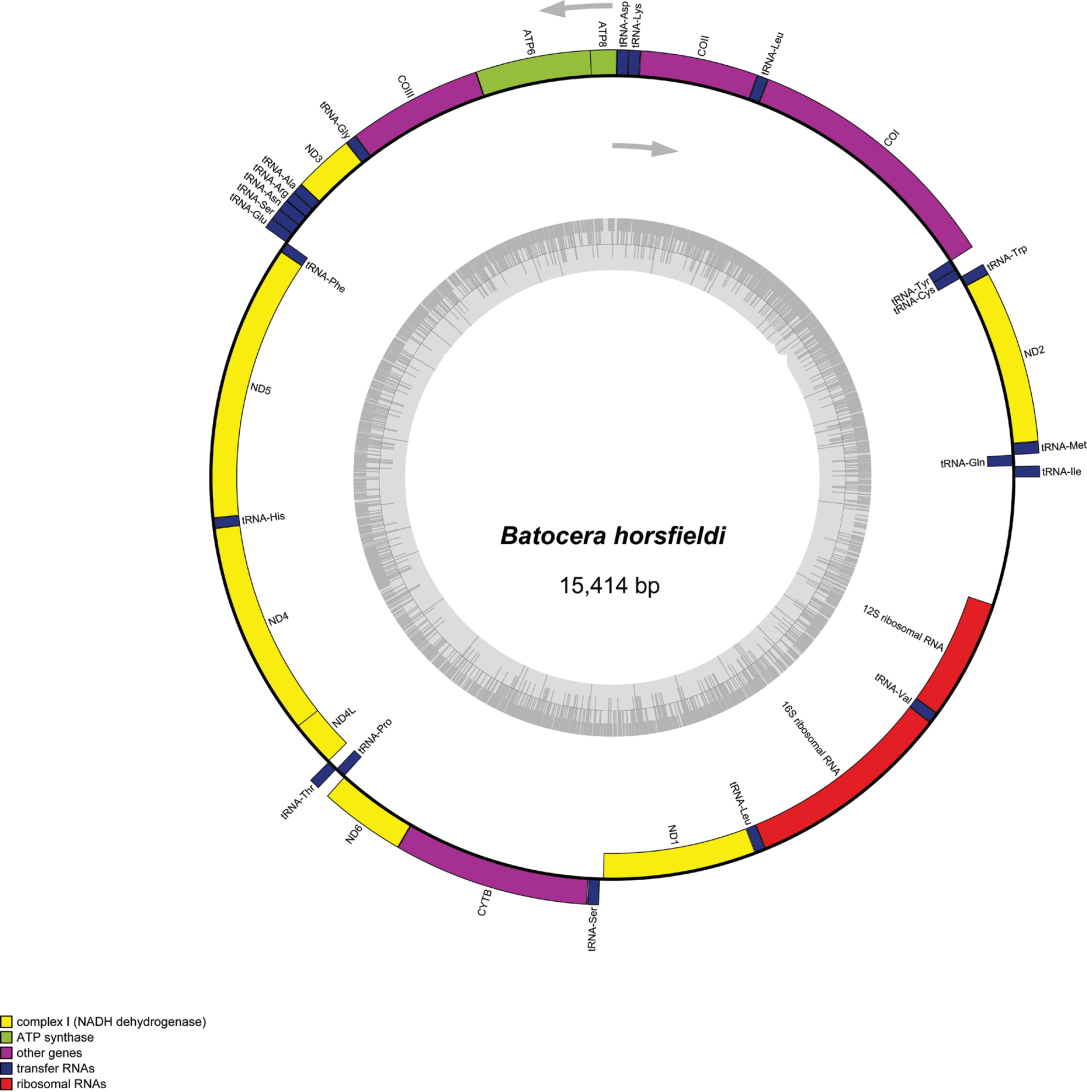
Mitogenome map of *B.horsfieldi*. ND1, ND2, ND3, ND4, ND4L, ND5 and ND6 are in yellow. COI, COII and COIII are in magenta. ATP6 and ATP8 are in green. CYTB is in pink. rrnl and rns are in red. all tRNAs are in dark blue and the control region is in white. Figure 1 was generated by Geneious v.11.0.2 by referring to Greiner et al. (2019).

The complete mitochondrial genome of *B.horsfieldi* is 15 425 bp in length and is a closed circular molecule (Fig. 1). It contains 37 genes, consisting of 22 transfer RNAs, two ribosomal RNAs, 13 protein-coding genes (PCGs), and an A+T-rich region, which is a feature shared with other typical Cerambycidae mitogenomes. Of the 13 PCGs, 9 (ND2, ND3, ND6, COI, COII, COIII, ATP8, ATP6, and CYTB) are encoded on the J strand, along with 14 tRNAs (Ile, Met, Trp, Leu2, Lys, Asp, Gly, Ala, Arg, Asn, Ser1, Glu, Thr, and Ser2), and a control region. The remaining four PCGs (ND5, ND4, ND4L, and ND1), eight tRNAs (Gln, Cys, Tyr, Phe, His, Pro, Leu1, and Val), and two rRNAs (12S rRNA and 16S rRNA) are encoded on the N strand (Fig. 1). The gene order of the new mitogenome follows the ancestral arrangement proposed for all arthropods (Braband et al. 2010). The overall mitogenome is composed of 33.12% A, 41.64% T, 12.08% C and 13.16% G, with a highly biased A+T content of 74.76% (Table 2). The calculation of asymmetrically distributed nucleotide content was determined by the formulas AT-skew = (A - T)/(A + T) and GC-skew = (G - C)/(G + C). The AT-skew and GC-skew of the *B.horsfieldi* mitogenome are -0.11 and 0.04, respectively (Table 2), indicating a negative AT-skew and a positive GC-skew. Overall, the nucleotide composition and genomic structure of *B.horsfieldi* exhibit typical features of the mitogenomes of insects in the order Coleoptera.

**Table 2.** Base composition in different regions of the mitochondrial genome of *B.horsfieldi*.

Region	Length (bp)	A%	T%	C%	G%	A+T%	AT-skew	GC-skew
ND1	951	27.44	44.58	8.31	19.66	72.02	-0.24	0.41
ND2	1011	33.14	40.36	18.30	8.21	73.50	-0.10	-0.38
ND3	354	31.36	43.22	16.38	9.04	74.58	-0.16	-0.29
ND4	1333	29.78	48.16	6.98	15.08	77.84	-0.24	0.37
ND4L	288	29.17	49.13	7.64	13.89	78.48	-0.26	0.29
ND5	1717	31.16	46.88	7.75	14.21	78.04	-0.20	0.29
ND6	504	37.7	42.86	13.29	6.15	80.56	-0.06	-0.37
ATP6	675	32.74	41.33	16.74	9.19	74.07	-0.12	-0.29
ATP8	156	42.95	44.23	8.97	3.85	87.18	-0.01	-0.40
COI	1543	30.46	35.84	18.79	14.91	66.30	-0.08	-0.12
COII	688	32.27	38.52	18.17	11.05	70.79	-0.09	-0.24
COIII	787	30.50	38.25	17.03	14.23	68.75	-0.11	-0.09
CYTB	1143	32.37	38.58	17.76	11.29	70.95	-0.09	-0.22
Rrnl	1283	34.53	39.91	7.17	18.39	74.44	-0.07	0.44
Rrns	751	33.95	43.14	7.46	15.45	77.09	-0.12	0.35
rRANs	2034	34.32	41.10	7.28	17.31	75.42	-0.09	0.41
tRNAs	1450	38.55	37.79	10.07	13.59	76.34	0.01	0.15
13PCGs	11150	31.42	42.13	13.60	12.85	73.55	-0.15	-0.03
A+T-rich region	791	43.99	43.11	6.83	6.07	87.10	0.01	-0.06
Whole Genome	15425	33.12	41.64	12.08	13.16	74.76	-0.11	0.04

### ﻿PCGs and codon usage

**Figure 2.**
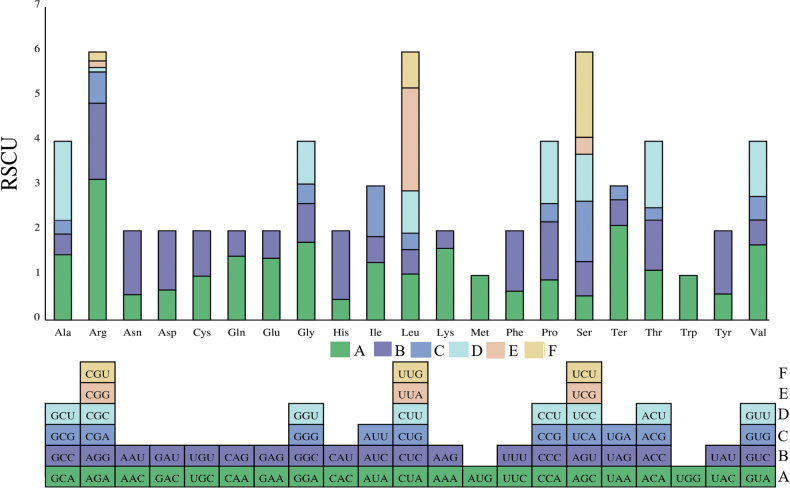
Relative synonymous codon usage (RSCU) of the obtained mitogenome. RSCU value represented on the y-axis, and the *B.horsfieldi* codons of their respective amino acids represe on the x-axis.

The total length of the 13 PCGs in the mitochondrial genome of *B.horsfieldi* is 11 150 bp, accounting for 72.29% of the entire genome. Among the 13 PCGs, four genes (ND4, ND4L, ND5, and ND1) are encoded on the N strand, while the other nine genes (COI, COII, COIII, ATP8, ATP6, NAD2, NAD3, NAD6, and CYTB) are encoded on the J strand. Among the 13 PCGs, the longest gene is COX1, but its A+T content is the lowest at 66.30%, while the shortest gene is ATP8, but its A+T content is the highest at 87.18%. The start codons for COI, COII, ATP8, ND5, and ND6 are ATT, while the start codon for ND1 is TTG. The start codons for COIII, ATP6, ND3, ND4, ND4L, and CYTB are ATN (N represents G or C) (Table 3). Seven of the 13 PCGs share the typical termination codons TAA and TAG, while CYTB uses CTC as the termination codon, and the remaining four PCGs use a single T residue as the termination codon (Table 3). It is widely believed that the incomplete codon structure signifies the stop of protein translation in insects and other invertebrates (Cheng et al. 2016). Besides, the calculation of relative synonymous codon usage (RSCU) values and the analysis of base composition bias are important references for studying the replication and transcription mechanisms of mitochondrial genomes (Wei et al. 2010). We summarized the usage of relative synonymous codons. The results showed that AGA, UUA, UAA, and UCU are the four most commonly used codons (Fig. 2).

**Table 3.** Characteristics of *B.horsfieldi* mitochondrial genome. J and N represent the positive and negative chains of mitochondrial genome, respectively.

Gene	Strand	Region	Length (bp)	Start codon	Stop codon	Anticodon
tRNA-Ile	J	1–68	68			GAU
tRNA-Gln	N	70–138	69			UUG
tRNA-Met	J	138–206	69			CAU
ND2	J	207–1217	1011	ATC	TAA	
tRNA-Trp	J	1216–1281	66			UCA
tRNA-Cys	N	1274–1336	63			GCA
tRNA-Tyr	N	1337–1402	66			GUA
COI	J	1395–2937	1543	ATT	T	
tRNA-Leu2	J	2938–3002	65			UAA
COII	J	3003–3690	688	ATT	T	
tRNA-Lys	J	3691–3759	69			UUU
tRNA-Asp	J	3760–3827	68			GUC
ATP8	J	3828–3983	156	ATT	TAG	
ATP6	J	3977–4651	675	ATG	TAA	
COIII	J	4651–5437	787	ATG	T	
tRNA-Gly	J	5438–5501	64			UCC
ND3	J	5502–5855	354	ATC	TAG	
tRNA-Ala	J	5854–5917	64			UGC
tRNA-Arg	J	5918–5979	62			UCG
tRNA-Asn	J	5979–6045	67			GUU
tRNA-Ser1	J	6046–6112	67			UCU
tRNA-Glu	J	6113–6176	64			UUC
tRNA-Phe	N	6176–6239	64			GAA
ND5	N	6240–7956	1717	ATT	T	
tRNA-His	N	7957–8019	63			GUG
ND4	N	8020–9352	1333	ATG	T	
ND4L	N	9346–9633	288	ATG	TAA	
tRNA-Thr	J	9636–9700	65			UGU
tRNA-Pro	N	9701–9765	65			UGG
ND6	J	9768–10271	504	ATT	TAA	
CYTB	J	10271–11413	1143	ATG	CTC	
tRNA-Ser2	J	11416–11483	68			UGA
ND1	N	11504–12454	951	TTG	TAG	
tRNA-Leu1	N	12456–12520	65			UAG
*rRNl*	N	12521–13803	1283			
tRNA-Val	N	13804–13872	69			UAC
rRNs	N	13873–14623	751			
Control region	J	14624–15414	791			

### ﻿tRNA genes, rRNA genes and A+T-rich region

The total length of the 22 tRNAs in *B.horsfieldi* is 1450 bp, ranging from 61 to 70 bp (Table 3). The nucleotide composition of the tRNAs is 38.55% A, 37.79% T, 10.07% C, and 13.59% G. The AT-skew and GC-skew are 0.01 and 0.15, respectively (Table 2). Eight of the 22 tRNA genes are located on the N strand, while the other 14 are located on the J strand. The tRNA secondary structure of *B.horsfieldi* was forecasted by the tRNAScan-SE server v.1.21.

**Figure 3.**
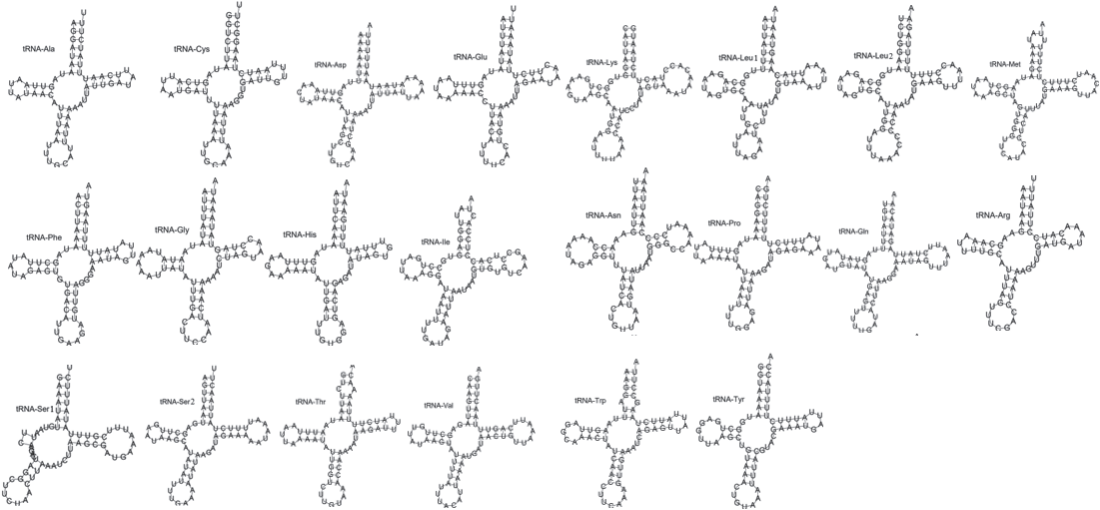
tRNAs secondary structure of *B.horsfieldi*.

The outcomes revealed that all tRNA genes conform to the standard cloverleaf structure, with the exception of tRNA-ser1, which is lacking a characteristic feature - the dihydrouridine arm (Fig. 3). The result is aligned with previous studies, such as those on stingray [*Acroteriobatusannulatus*, *Acroteriobatusblochii* (Van Staden et al. 2022)] and beetles [*Coomaniellacopipes*, *Coomanielladentata* and *Dicercacorrugata* (Huang et al. 2022)]. Moreover, the lengths of the 16S rRNA and 12S rRNA genes are 1283 bp and 751 bp, respectively (Table 2). The 16S rRNA is located between tRNA-Leu and tRNA-Val and is 1283 bp, constituting 34.53% A, 39.91% T, 7.17% C, and 18.39% G. The 12S rRNA is located between tRNA-Val and the A+T-rich region and is 751 bp in length. The nucleotide composition of the 12S rRNA is 33.95% A, 43.14% T, 7.46% C, and 15.45% G. The AT-skew and GC-skew of the RRNs are -0.09 and 0.41, respectively (Table 2). Compared with other longhorn beetles, the positions and characteristics of the 16S rRNA and 12S rRNA genes are similar. The A+T-rich region of *B.horsfieldi* is located between trnI and rns genes and is 791 bp in length, with an A+T content of 87.1%, an AT-skew of 0.01, and a GC-skew of -0.06 under normal conditions.

### ﻿Phylogenetic analysis

**Figure 4.**
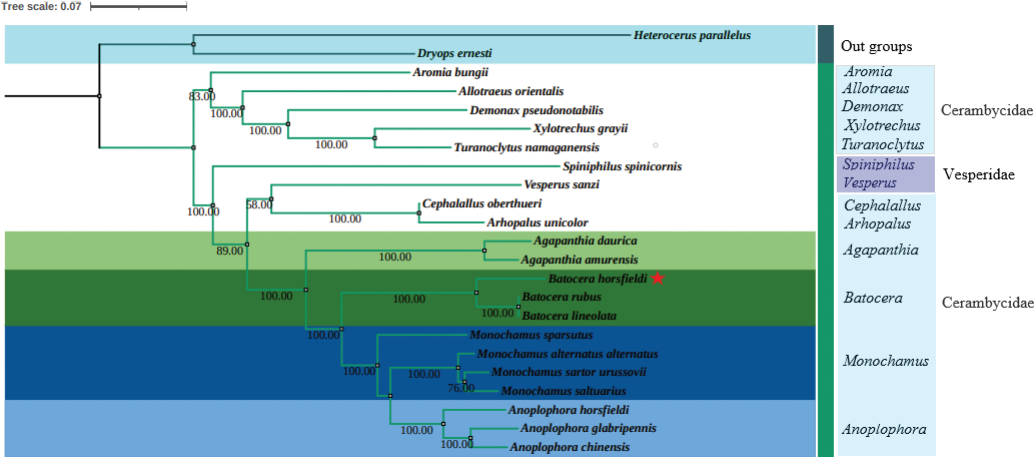
Phylogenetic relationships of 21 selected longhorn beetles and outgroups (*Heterocerusparallelus* and *Dryopsernesti*) inferred based on maximum likelihood of 13 protein-coding genes and two rRNA genes. Values less than 70 are not displayed.

**Figure 5.**
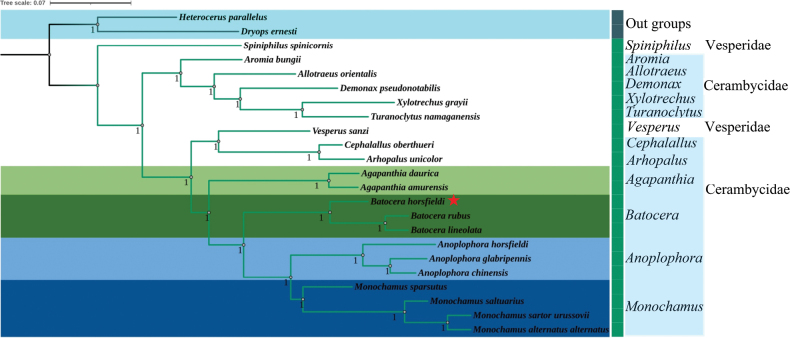
Phylogenetic relationships of 21 selected longhorn beetles and outgroups (*Heterocerusparallelus* and *Dryopsernesti*) inferred based on Bayesian analyses of 13 protein-coding genes and two rRNA genes. Numbers on branches are posterior probabilities.

Thirteen protein-coding genes (PCGs) and two ribosomal RNA (rRNA) sequences were utilized to construct the phylogenetic trees of *B.horsfieldi* via maximum likelihood (ML) and Bayesian inference (BI) methods (Figs 4, 5). Based on the results obtained from BI, the monophyly of Cerambycidae was once again confirmed, as all the species belonging to this family formed a highly supported single clade. However, the results obtained from ML showed that Cerambycidae and Vesperidae formed a highly supported single clade. In both BI and ML, the two outgroups were clearly separated from the Cerambycidae and Vesperidae clades. Interestingly, BI showed that *B.horsfieldi*, *B.rubus* and *B.lineolata*, along with four species of *Monochamus* and three species of *Anoplophora*, were clustered into a branch with high node support values. On the other hand, ML showed that *B.horsfieldi*, *B.rubus* and *B.lineolata* were only clustered with *Monochamussparsutus* Fairmaire, 1889 (*M.sartorurussovii* Fischer-Waldheim, 1806 +*M.saltuarius* Gebler, 1830) +*M.alternatusalternatus* Hope, 1842), and (*Anoplophorachinensis* Forster, 1771 +*A.glabripennis* Motschulsky, 1853) +*A.horsfieldi* Hope, 1842), all of which had high node support values. Additionally, ML showed that Vesperidae was only clustered with seven genera of Cerambycidae (*Cephalallus*, *Arhopalus*, *Agapanthia*, *Batocera*, *Monochamus*, and *Anoplophora*), all with high node support values, whereas BI showed that Vesperidae was clustered with 13 genera of Cerambycidae used in this study, all with high node support values. Furthermore, BI showed that *Spiniphilusspinicornis* Lin & Bi, 2011 (Vesperidae), *Vesperussanzi* Reitter, 1895 (Vesperidae)and the 19 Cerambycidae species used in this study formed a sister clade.

## ﻿Discussion

The complete mitochondrial genome sequence of *B.horsfieldi* obtained in this work enriches the species data of *Batocera*, Cerambycidae. Comparative analysis and phylogenetic analysis of the sequence features of Cerambycidae mitochondrial genomes were carried out by combining 13 protein-coding genes (PCGs) and two ribosomal RNA (rRNA) data from 21 species of longhorn beetles publicly available on NCBI. The results of this study will lay the foundation for population genetics research on *B.horsfieldi* and phylogenetic reconstruction of the Cerambycidae.

The secondary structures of tRNAs in Cerambycidae mitochondrial genomes have a typical cloverleaf structure, except for the absence of the D-arm in tRNA-ser1 (AGN) (Yang et al. 2017). The absence of the D-arm in trnS1 (AGN) is commonly found in other animal and insect orders, such as Lepidoptera, Diptera and Hemiptera (Wolstenholme 1992; Liao et al. 2010). Based on the start and stop codons of the protein-coding regions, most of the mitochondrial protein-coding genes in Cerambycidae begin with the typical ATN start codon, with only a few species of ND1 having a special start codon TTG, and the stop codons are generally the common TAN and incomplete T codons (Yang et al. 2017). Incomplete stop codons (T or TA) are common in animal mitochondrial genomes (Boore 1999) and Coates et al. (2005) suggest that they can be completed to TAA by the A at the 3’ end of the transcript. There has been ongoing controversy regarding the start codon for COI, and the use of different start or stop codons can result in variations in the number of base pairs between genes (Nie and Yang 2014). According to Sheffield et al. (2008), the start codon for COI in the suborder Polyphaga is either AAT or AAC; however, a reannotation was conducted in this study, which revealed that ATT is the start codon for COI in *B.horsfieldi*. Interestingly, in a recent study (Mei et al. 2022), the start codon for COI was reported to be ATG, which is similar to our findings. The A+T-rich region is the most important non-coding region in mitochondrial genomes, with extremely high A+T content. Length variation in this region can be very large, even among closely related species (Zhang et al. 1995; Zhang and Hewitt 1997). *Batocerahorsfieldi* has an A+T-rich region of 791 bp, which results in a significantly longer mitochondrial genome compared to other genomes.

Currently, traditional taxonomic studies of longhorn beetles are mainly based on morphological features; however, there are still many controversies due to the complexity and instability of the morphological features. In this study, a phylogenetic tree was constructed using BI and ML methods based on 13 PCGs and 2 rRNAs, exhibiting differences with previous related studies. For instance, our results suggest that *A.glabripennis* and *A.chinensis* belong to the same branch, whereas in the study by Li et al. (2011), they were not grouped together. Furthermore, our findings indicate a closer relationship between *M.sartorurussovii* and *M.alternatusalternatus*, which contrast with the results of the study conducted by Yang et al. (2017). This work aimed to analyze the phylogenetic relationships of 21 longhorn beetle species and two outgroups (*H.parallelus* and *D.ernesti*) using mitochondrial genome data. Although the ML and BI trees constructed from 13 PCGs and two rRNA sequences did not exhibit identical topologies, their expression results were quite similar. Notably, the node support values of BI trees were consistently higher than those of ML trees for the same dataset, which has been observed in many previous studies of other taxa (Yuan et al. 2016; Chen et al. 2018; Li et al. 2019). Our results indicate that mitochondrial genome sequences are useful tools for inferring relationships among Cerambycidae. Furthermore, the technological development of sequencing and assembly of mitochondrial genomes will facilitate future work in mitochondrial genome sequencing (Crampton-Platt et al. 2015; Bourguignon et al. 2017). Increasing the sampling of taxa and genome sequencing will further help resolve the classification issues of longhorn beetles. Although this study has contributed one new complete mitochondrial genome to the phylogeny of Cerambycidae, the interrelationships among subfamilies and tribes still require more data to be completely determined. These questions will be well addressed in the future when sufficient numbers of complete mitochondrial genomes of longhorn beetles are accumulated. Although our study data cannot fully resolve the phylogenetic relationships within Cerambycidae, it provides a reference for further research on the phylogeny of Cerambycidae.

## ﻿Conclusions

In this study, we successfully obtained the complete mitochondrial genome of *Batocerahorsfieldi* for the first time, which is 15 425 bp in length. The mitochondrial genome of *B.horsfieldi* exhibits a molecular pattern similar to that of other Cerambycidae species, comprising 37 gene segments, including 22 transfer RNAs, two ribosomal RNAs, 13 protein-coding genes (PCGs), and an A + T-rich region typical of other Cerambycidae mitogenomes. Four of the 13 PCGs are encoded on the N strand, while the remaining nine genes are encoded on the J strand. The start codons of COI, COII, ATP8, ND5, and ND6 are ATT, while that of ND1 is TTG, and the start codons of COIII, ATP6, ND3, ND4, ND4L, and CYTB are ATN (N represents G or C). Seven of the 13 PCGs share typical stop codons (TAA and TAG), while CYTB uses CTC as a stop codon and the remaining four PCGs use a single T residue as a stop codon. All tRNAs exhibit typical cloverleaf structures except for tRNASer1. Phylogenetic analyses revealed that Cerambycidae formed a highly supported single clade, and Vesperidae was either clustered with Cerambycidae or formed a separate clade. Interestingly, *B.horsfieldi*, *B.rubus* and *B.lineolata* were clustered with *Monochamus* and *Anoplophora* species in both analyses, with high node support values. Additionally, *Spiniphilusspinicornis* and the 20 longhorn beetles formed a sister clade in the Bayesian analysis.

## ﻿Additional information

### Author ORCIDs

Zhihang Zhuo https://orcid.org/0000-0002-2566-3172

### Data availability

The mitogenomes were deposited at Figshare. https://doi.org/10.6084/m9.figshare.22666192.v1 and https://doi.org/10.6084/m9.figshare.22673647.v1.

## Supplementary materials

Supplementary material 1 *Batocerahorsfieldi* and 22 species Data type Phylogenetic (docx. file) https://binary.pensoft.net/file/872891This dataset is made available under the Open Database License (http://opendatacommons.org/licenses/odbl/1.0/). The Open Database License (ODbL) is a license agreement intended to allow users to freely share, modify, and use this Dataset while maintaining this same freedom for others, provided that the original source and author(s) are credited.Junhao Wu,Zhihang Zhuo
