# Supplementary material for: ﻿Characterization of the complete mitochondrial genome of the longhorn beetle, Batocerahorsfieldi (Coleoptera, Cerambycidae) and its phylogenetic analysis with suitable longhorn beetles
Source: Zookeys. 2023 Jul 4;1168:387–402. doi: 10.3897/zookeys.1168.105328 (PMC10336557; doi:10.3897/zookeys.1168.105328)
Supplement: Supplementary material 1 — Batocerahorsfieldi and 22 species [file zookeys-1168-387_article-105328__-s001.docx]

Supplementary File

For: Characterization of the complete mitochondrial genome of the longhorn beetle, *Batocera* *horsfieldi* (Coleoptera, Cerambycidae), and its phylogenetic analysis with suitable longhorn beetles

**1. Adult image**


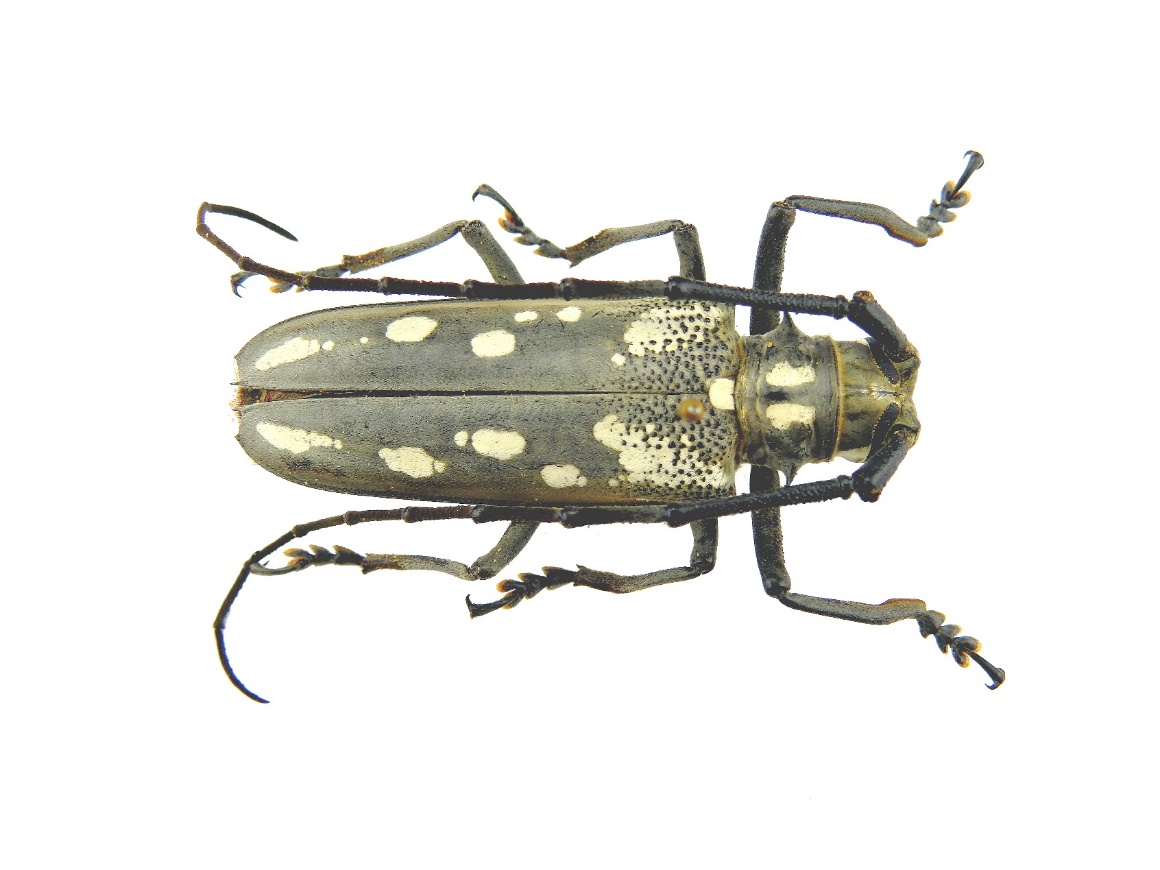


***Batocera horsfieldi* (Male, adult)**

**2. Insect identification materials**

1) Hua LZ,Nara Hajime,G.A.Saemulson,S.W.Lingafelter(2009) Iconography of Chinense Longicorn Beetles (1406 Species) in Color. Sun Yat - sen University Press, Guangzhou,69-70pp


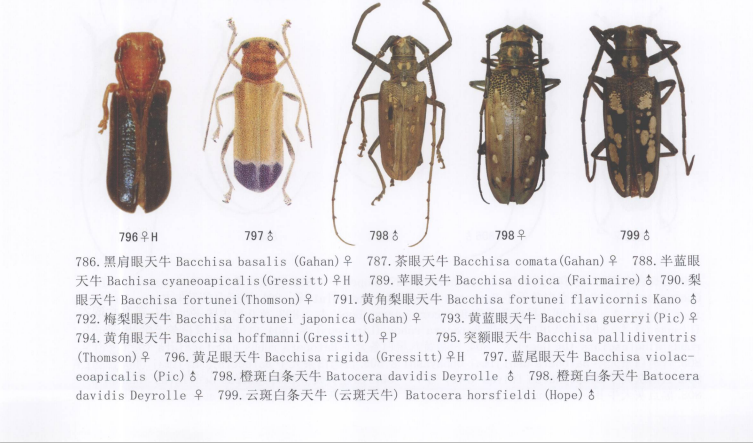


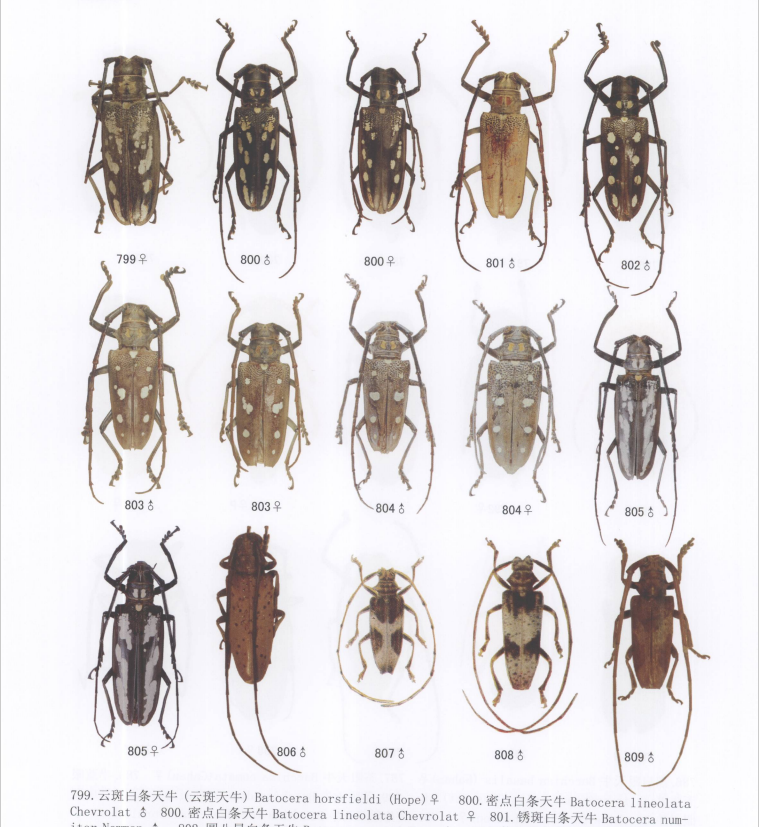


2) Liu Y, Xiong S, Ren JQ, Zhang XX, Chen L. (2012) Comparative morphological study on genus *Batocera* (Coleoptera, Cerambycidae, Lamiinae, Batocerini). Acta Zootaxonomica Sinica, 37(04): 701-711.


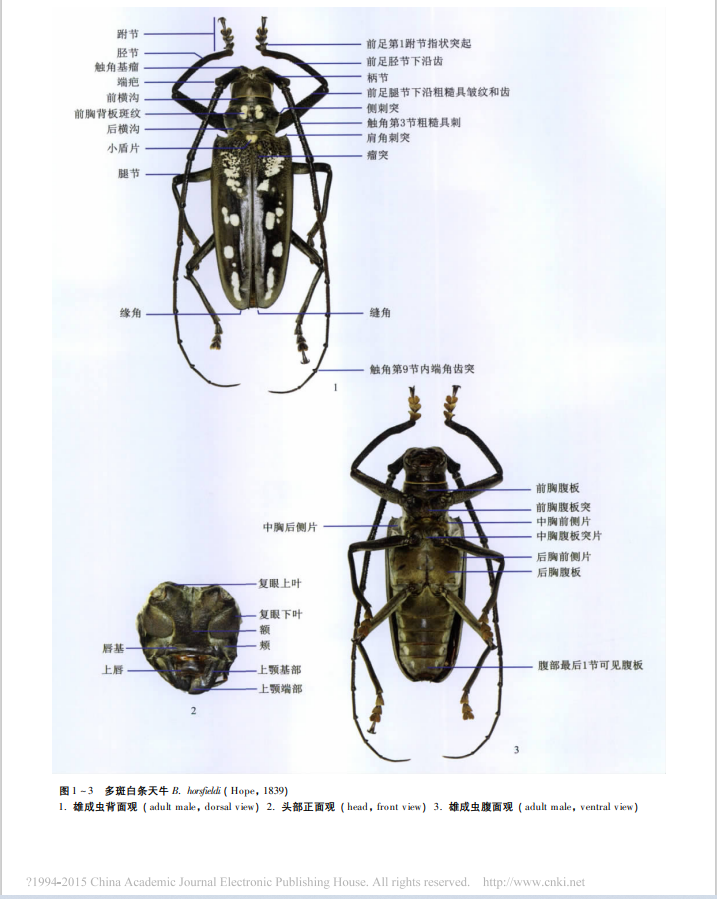


**3. Data availability**

For B. horsfieldi: https://doi.org/10.6084/m9.figshare.22666192.v1.

For 22 species: https://doi.org/10.6084/m9.figshare.22673647.v1 .

Table S1 GenBank accession numbers of species used in this study.

| Family | Genus | Species | GenBank  Accession  number |
| --- | --- | --- | --- |
| Cerambycidae | *Monochamus* | [*Monochamus sparsutus*](https://www.ncbi.nlm.nih.gov/nuccore/NC_053906.1) | NC053906.1 |
|  |  | [*Monochamus alternatus*](https://www.ncbi.nlm.nih.gov/nuccore/NC_050066.1) | NC050066.1 |
|  |  | [*Monochamus sartor*](https://www.ncbi.nlm.nih.gov/nuccore/OP856519.1) *urussovii* | OP856519.1 |
|  |  | [*Monochamus saltuarius*](https://www.ncbi.nlm.nih.gov/nuccore/OP169419.1) | OP169419.1 |
|  | *Agapanthia* | *Agapanthia amurensis* | MW617354.1 |
|  |  | *Agapanthia daurica* | MN473114.1 |
|  | *Anoplophora* | *Anoplophora glabripennis* | NC008221.1 |
|  |  | *Anoplophora horsfieldi* | MW364565.1 |
|  |  | [*Anoplophora chinensis*](https://www.ncbi.nlm.nih.gov/nuccore/NC_029230.1) | NC029230.1 |
|  | *Batocera* | *Batocera lineolata* | MW629558.1 |
|  |  | *Batocera rubus* | OM161963.1 |
|  |  | *Batocera horsfieldi* | **OQ785650** |
|  | *Xylotrechus* | [*Xylotrechus grayii*](https://www.ncbi.nlm.nih.gov/nuccore/NC_030782.1) | NC030782.1 |
|  | *Turanoclytus* | [*Turanoclytus namaganensis*](https://www.ncbi.nlm.nih.gov/nuccore/NC_060874.1) | NC060874.1 |
|  | *Demonax* | [*Demonax pseudonotabilis*](https://www.ncbi.nlm.nih.gov/nuccore/OP096419.1) | OP096419.1 |
|  | *Allotraeus* | [*Allotraeus orientalis*](https://www.ncbi.nlm.nih.gov/nuccore/NC_061181.1) | NC061181.1 |
|  | *Arhopalus* | [*Arhopalus unicolor*](https://www.ncbi.nlm.nih.gov/nuccore/NC_053904.1) | NC053904.1 |
|  | *Aromia* | [*Aromia bungii*](https://www.ncbi.nlm.nih.gov/nuccore/NC_053714.1) | NC053714.1 |
|  | *Cephalallus* | [*Cephalallus oberthueri*](https://www.ncbi.nlm.nih.gov/nuccore/NC_062854.1) | NC062854.1 |
|  | *Vesperus* | *Vesperus sanzi* | MN473093.1 |
| Vesperidae | *Spiniphilus* | [*Spiniphilus spinicornis*](https://www.ncbi.nlm.nih.gov/nuccore/NC_029515.1) | NC029515.1 |
| Out groups | *Dryops* | *Dryops ernesti* | KX035147.1 |
|  | *Heterocerus* | *Heterocerus parallelus* | KX087297.1 |
